# Optimizing ultrashort pulse in fiber laser based on artificial intelligence algorithm

**DOI:** 10.1038/s41598-024-58630-6

**Published:** 2024-04-04

**Authors:** Xiaoxiang Han, Zhiting Huang, Jun Yue, Jun Li, Xiang’an Yan, Yanwen Xia, Guoqing Zhang, Haiyang Zhang, Caijuan Xia, Yusheng Zhang

**Affiliations:** 1 School of Science, Xi’an Polytechnic University, Xi’an, 710048 Shaanxi China; 2 Research Center of Laser Fusion, CAEP, Mianyang, 621900 China; 3 Hangzhou Institute of Advanced Studies, Zhejiang Normal University, Hangzhou, 311231 China; 4 Engineering Research Center of Flexible Radiation Protection Technology, Universities of Shaanxi Province, Xi’an Polytechnic University, Xi’an, 710048 Shaanxi China; 5 Xi’an Key Laboratory of Nuclear Protection Textile Equipment Technology, Xi’an Polytechnic University, Xi’an, 710048 Shaanxi China

**Keywords:** Lasers, LEDs and light sources, Optical techniques

## Abstract

Ultrashort pulses, characterized by their short pulse duration, diverse spectral content, and high peak power, are widely used in fields including laser processing, optical storage, biomedical sciences, and laser imaging. The complex, highly-nonlinear process of ultrashort pulse evolution within fiber lasers is influenced by numerous aspects such as dispersion, loss, gain, and nonlinear effects. Traditionally, the split-step Fourier transforms method is employed for simulating ultrashort pulses in fiber lasers, which involves traversing multiple parameters within the fiber to attain the pulse’s optimal state. The simulation is a significantly time-consuming process. Here, we use a neural network model to fit and predict the impact of multiple parameters on the pulse characteristics within fiber lasers, enabling parameter optimization through genetic algorithms to determine the optimal pulse duration, pulse energy, and peak power. Integrating artificial intelligence algorithms simplifies the acquisition of optimal pulse parameters and enhances our understanding of multiple parameters’ impact on the pulse characteristics. The investigation of ultrashort pulse optimization based on artificial intelligence holds immense potential for laser design.

## Introduction

Fiber lasers, characterized by their superior beam quality, high stability, and compact structure^1,2^, are instrumental in fields including optical communications^3^ and precision manufacturing^4–6^. The generation of disparate ultrashort pulses can be traced back to the laser resonant cavity’s unique parameter settings. These settings can be optimized to yield a variety of ultrashort pulses, fulfilling the demands of diverse application fields. The traditional simulation for pulse evolution in fiber lasers are usually based on the nonlinear Schrödinger equation (NLSE) and the split step Fourier transform (SSFT) method^7,8^. However, due to the multitude of factors which influence pulse characteristics within the laser resonant cavity, traditional methods usually require numerous adjustments to the resonant cavity’s parameters based on the researcher's experience, to pinpoint the optimal operational state of the laser outputting the desired pulse. This approach, while thorough, necessitates substantial time to adapt to differing resonant cavity outputs and compromises on efficiency. Hence, selecting components with suitable parameters to produce the desired target pulse is a pressing challenge in fiber laser design.

The swift development of artificial intelligence (AI) technology has prompted researchers to explore efficient, convenient avenues for information acquisition and processing^9,10^. Genty et al*.* have reviewed the rapid growth and development of the field of smart photonics, where machine-learning (ML) algorithms are being matched to optical systems to add new functionalities and to enhance performance^11^. Although the human brain’s thought architecture is somewhat intricate, the combination of hardware and software, supported by robust algorithms, manages to simulate or supplant certain aspects of human behavioral and thought patterns, achieving remarkable learning capabilities^12,13^. AI optimization algorithms autonomously generate functions accomplishing tasks tailored to human needs, learning from data samples, processing information through programming, and eventually accomplishing the assigned target^14^. Owing to the impressive learning rates of such optimization algorithms, they have been introduced into many fields^15–19^. Lately, the combination of laser technology and AI algorithms has been increasingly utilized in the optimization of mode-locked fiber lasers^20–22^. Yu et al*.* have highlighted the areas where AI exhibits potential in accelerating the development of mode-locked fiber lasers, including nonlinear dynamics prediction, ultrashort pulse characterization, inverse design, and automatic control of mode-locked fiber lasers^23^. Rapid identification of the laser's optimal operational state, facilitated by these AI algorithms, leads to an efficient optimization process. This method has higher efficiency and accuracy compared to traditional empirical methods, which can significantly improve optimization effectiveness.

In this paper, we employ a neural network (NN) and genetic algorithm (GA) to find the laser cavity’s optimal parameter settings in terms of pulse duration, pulse energy, and peak power. The NN is used to fit and predict the impact of multiple parameters on ultrashort pulses in laser cavity, followed by the deployment of a GA to identify optimal pulse duration, energy, and peak power values. The advantage of this method is that it can quickly and effectively comprehensively evaluate the influence of multiple parameters on the ultrashort pulse characteristics, eliminating the need for extensive experiments and complex theoretical analysis. It also offers invaluable insight for the design and optimization of various parameters affecting pulses, which contributes to the formulation of laser schemes. The investigation of ultrashort pulse based on AI paves the way for potential applications in laser precision machining.

## Results

### Setup of simulated laser cavity

**Figure 1:**
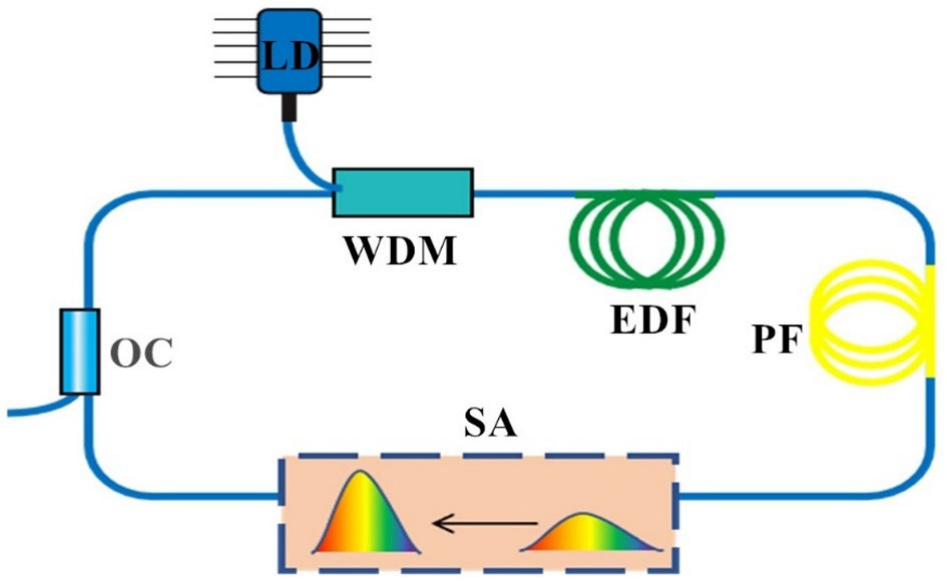
Schematic diagram of simulated laser cavity. LD, laser diode; WDM, wavelength division multiplexer; EDF, Erbium-doped fiber; PF, passive fiber; SA, saturable absorber; OC, optical coupler.

The analysis of pulse formation in fiber lasers based on NNs commences with the preparation of training samples. Here, SSFT is utilized to generate these training samples. Each individual sample encapsulates laser cavity parameters and output pulse information. The configuration of the laser utilized for simulation is depicted in Fig. 1, which is consist of a saturable absorber (SA), a section of passive fiber (PF) and an erbium-doped fiber (EDF) in the laser cavity. The selected laser employs a Gaussian pulse with a central wavelength of 1550 nm as the seed pulse and undertakes the simulation under normal dispersion conditions by modifying different laser cavity parameters. The intensity of input Gaussian pulse is very weak, which can be considered as noise pulse. The pulse within the laser cavity is programmed to repeat 1000 times, with a 5 picosecond (ps) time window set up to record the dynamic behavior of ultrashort pulses. Eventually, a stable pulse is produced.

The transmission of optical pulses within EDF can be expressed through the NLSF, which can be given as^5^:

1 $$\frac{\partial A}{{\partial z}} = - \frac{{i\beta_{2} }}{2}\frac{{\partial^{2} A}}{{\partial t^{2} }} + i\gamma |A|^{2} A + \frac{g}{2}A\left( {1 + \frac{1}{{\Omega_{g}^{2} }}\frac{{\partial^{2} }}{{\partial t^{2} }}} \right),$$

where *A* represents the slowly changing amplitude of optical pulse; *z* represents the transmission distance; *β*_2_ represents the group velocity dispersion; *γ* represents the nonlinear coefficient; *Ω* represents the gain bandwidth of EDF,* g* represents the gain coefficient of EDF^24^,

2 $$g = g_{0} \left( z \right)\exp \left( { - \int {|A|^{2} } dt/E_{s} } \right),$$

where *g*_0_ represents the small signal gain coefficient of EDF, *E*_s_ represents the saturable energy of EDF.

Here, we employ SSFT for sample simulation, which simulates the propagation process at each step by dividing the entire propagation distance into multiple shorter steps. At each step size, Fourier transform is used for signal conversion from the temporal domain to the frequency domain, addressing linear and non-linear effects, then converting the processed signal back to the temporal domain via an inverse Fourier transform. Through SSFT, an accurate simulation of optical pulse transmission and evolution in nonlinear fiber media is feasible^25^. The length of passive fiber and EDF are both set as 6 m. The output ratio of OC is set as 10%. The SSFT simulation shows that OC has a relatively small impact on the pulse duration and the growth of *g*_0_ will broaden the pulse duration and spectral width. Within the parameter range defined in Table 1, we utilized SSFT to generate a parameter dataset for the laser cavity, encompassing seven key parameters: gain bandwidth of EDF (*Ω*), gain saturable energy of EDF (*E*_s_), nonlinearity coefficient of EDF (*γ*_EDF_), nonlinearity coefficient of PF (*γ*_PF_), modulation depth of SA (*L*_0_), saturable absorber power of SA (*P*_sat_), and small signal gain coefficient of EDF (*g*_0_). Simultaneously, information regarding the stable pulse within the laser cavity, including pulse duration, pulse energy, and peak power, is recorded. The dataset then serves as resources to construct and train NNs for simulating and predicting the pulse dynamics within the laser cavity. During the sample preparation stage, data pertaining to single pulses alone is recorded. On account of the convergence criteria applied being incompatible for fitting multi-pulse situations, the dataset excludes multi-pulse state data.

**Table 1:** Range of laser cavity parameters.

Parameters	Lower limits	Upper limits	Unit
*Ω*	20	60	nm
*E*_S_	4 × 10^–11^	2 × 10^–11^	J
*γ*_EDF_	1 × 10^−3^	5 × 10^−3^	1/(W m)
*γ*_PF_	1 × 10^−3^	5 × 10^−3^	1/(W m)
*L*_0_	0.2	0.9	–
*P*sat	10	100	W
*g*_0_	1	10	dB/m

1000 samples were generated via SSFT. Each sample consists of 10 data. These data are seven laser cavity parameters and three output pulse parameters (i.e. pulse duration, pulse energy, and peak power). The seven laser cavity parameters are tunning parameters, and the three output parameters are the dependent variables. These samples were allocated to the training, validation, and testing sets with ratios of 0.7, 0.15, and 0.15, respectively. The training set updates the NN parameters, the validation set counters overfitting, and adjusts the network's hyperparameters, while the test set assesses the trained NN's performance. During the training process, MSE was used to evaluate the mean square error between predicted and actual values. MSE can consider uncertainty when training NNs and effectively prevent overfitting problems. The NN ultimately uses regression graphs to evaluate the fitting effect, where each data point has a corresponding position on the regression graph, where the x coordinate represents the target value (sample result) and the y coordinate represents the output value obtained by the NN. The closer all coefficient points are to the straight-line y = x, the better the fitting effect of the NN. By using NNs for fitting analysis, we can generate function programs related to the nonlinear relationship between the seven laser cavity parameters and pulse information. At the same time, this program can predict the pulse information of the randomly generated seven laser cavity parameters.

**Figure 2:**
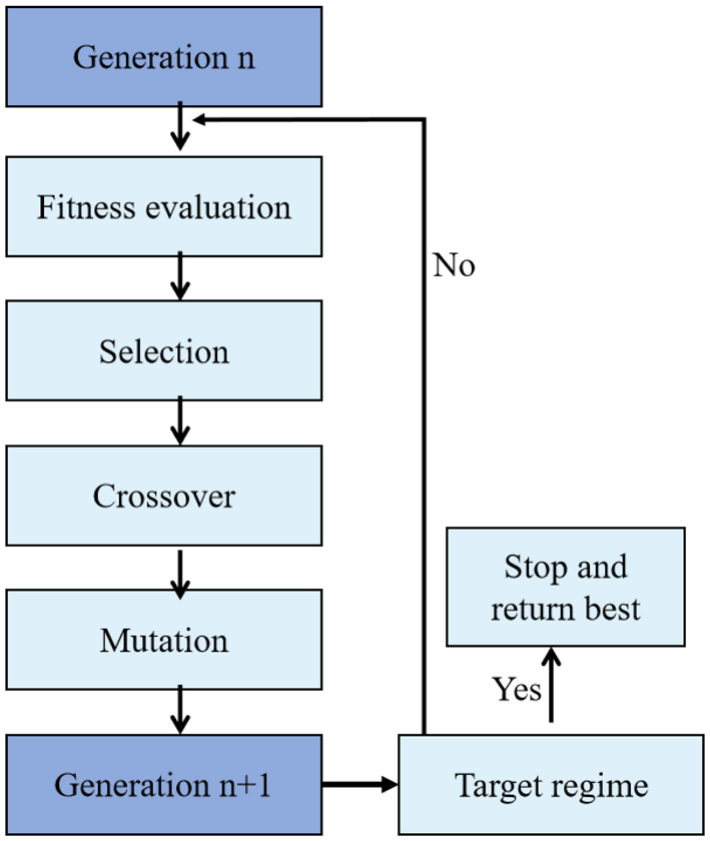
Genetic algorithm (GA) flowchart.

To locate the extremum of the mode-locked fiber laser, we adopt a GA along with a fitting function generated by an NN. The GA principle, as shown in Fig. 2, is based on the notion of natural selection, where only the fittest individuals survive the genetic process. In the GA, each individual represents an output pulse state, and their genes consist of seven laser cavity parameter values^26,27^, which means that each individual in GA is equivalent to one sample. Firstly, we randomly create an initial population of individuals within the range given in Table 1. Then, we test these individuals and evaluate their fitness based on the nonlinear functions generated by the NN. Next, select the individual with the highest score from the initial population as the parent of the gene, generate the next generation through crossover and mutation, and form a new population. Then, we compare the new generation of individuals with their parents, and if we find that the new generation of individuals is closer to the target than the previous individuals, we stop calculating. In the end, we successfully implement NNs and GAs to search for the extremum of mode-locked fiber lasers, thereby achieving optimal performance.

### Simulation results

The sample used for AI algorithm consists of seven laser cavity parameters and three output parameters (i.e. pulse duration, energy, and peak power). The number of samples in sample set is 1000, which and is generated by SSFT method. We initiated with the construction of the first NN with the output layer symbolizing the pulse duration. The mean squared error (MSE) of a NN is defined as the average square of the difference between the predicted value and the actual value. Assuming that the predicted output of the NN is *Y* and the actual value is *T*, MSE can be expressed as:

3 $$MSE = \frac{1}{N}\sum\limits_{i = 1}^{N} {(Y_{i} - T_{i} )^{2} }$$

**Figure 3:**
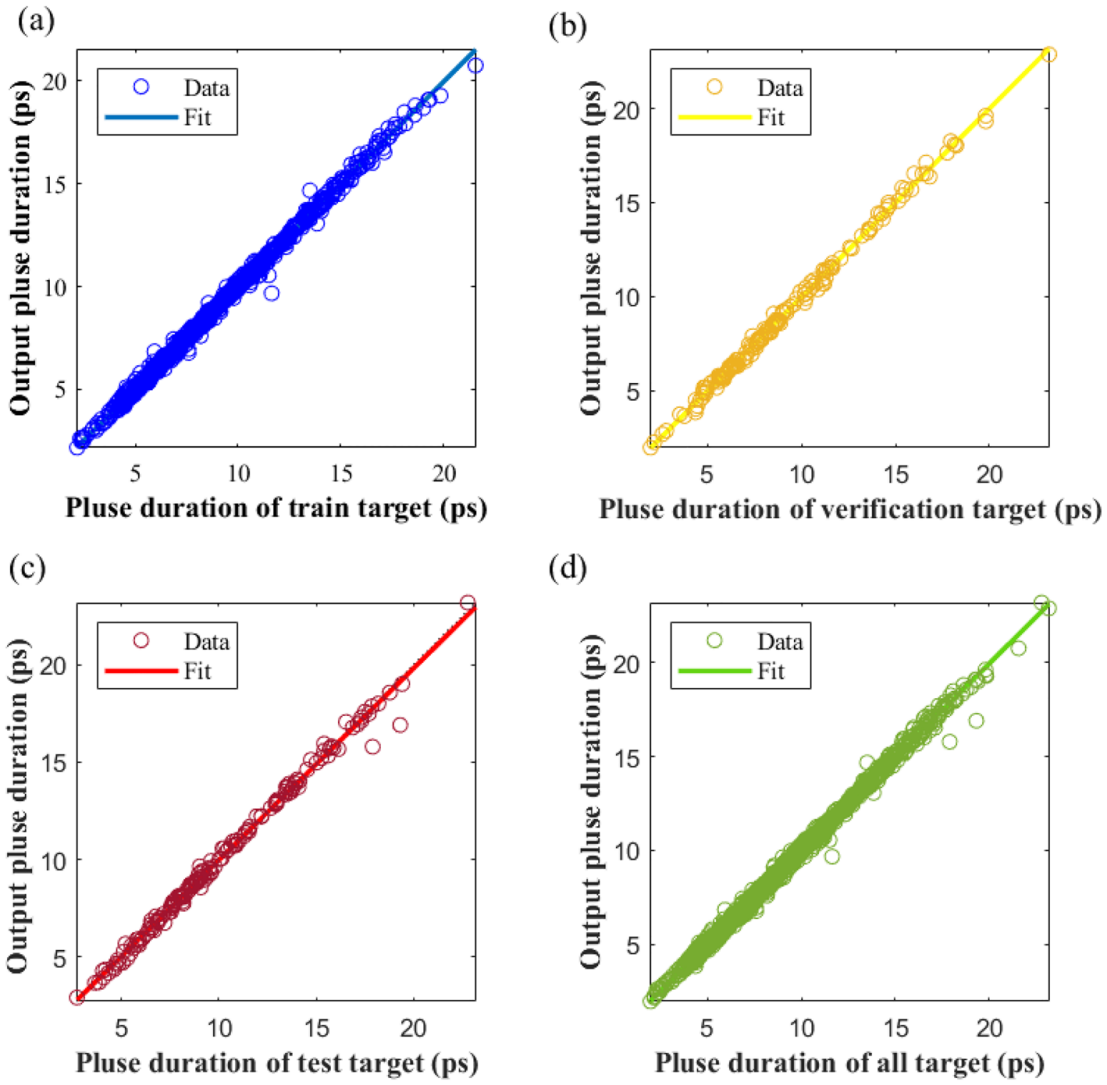
Comparative analysis of predicted and target values of pulse duration generated by neural networks (NNs). (**a**) Training dataset regression diagram, (**b**) validation dataset regression diagram, (**c**) test dataset regression diagram, (**b**) regression diagram of all dataset.

Among them, *N* is the number of samples, *Y*_i_ is the predicted value of the *i*_th_ sample, and *T*_i_ is the actual value of the *i*_th_ sample. The MSE for this model is calculated as 0.0419 for the training set, 0.0446 for the validation set, 0.1060 for the test set, and 0.0520 for the overall dataset. As can be deciphered from Fig. 3, the regression coefficients for the training set, validation set, test set, and the complete dataset are 99.83%, 99.861%, 99.68%, and 99.815% respectively. The correlation between the target value and the predicted value across all dataset exceeds 99%, and the fitting curve in each graph nearly coincides with y = x. This evidence indicates that the NN delivered commendable fitting performance on the training, validation, and testing sets.

**Figure 4:**
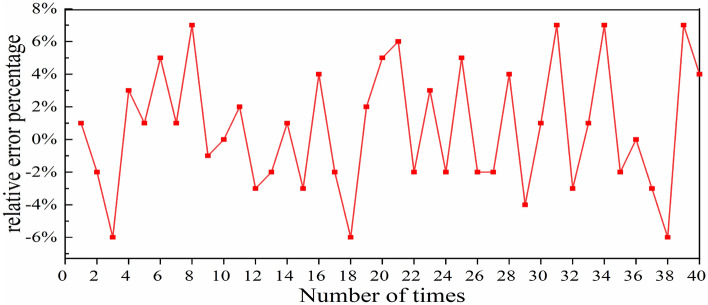
Relative percentage variation in pulse duration between the NN and SSFT set against the varying number of times.

The accuracy of the fitting function generated by the NN was put to test by employing 40 sets of randomly generated data. Each parameter value has an equal chance of being selected, and each parameter value is independent from each other. Through the NN, we can obtain 40 sets of output results. Another 40 sets of output results can be obtained by inputting these 40 sets of input parameters into SSFT. We compared the predicted pulse duration by the NN and the pulse duration computed using the SSFT. Figure 4 demonstrates the relative difference in the pulse’s duration obtained by two methods, which is calculated using the following formula:

4 $$\Delta = \frac{{\tau_{NN} - \tau_{SSFT} }}{{\tau_{SSFT} }} \times 100\% ,$$

where *τ*_NN_ is the pulse’s duration predicted by the NN, *τ*_SSFT_ is the pulse’s duration generated by SSFT. From Fig. 4, it can be observed that the maximum relative error between the two methods is 7%, illustrating that the pulse’s duration foreseen by the NN algorithm aligns well with the SSFT method. This evidence indicates that the NN delivered commendable fitting performance on the training, validation, and testing sets.

**Figure 5:**
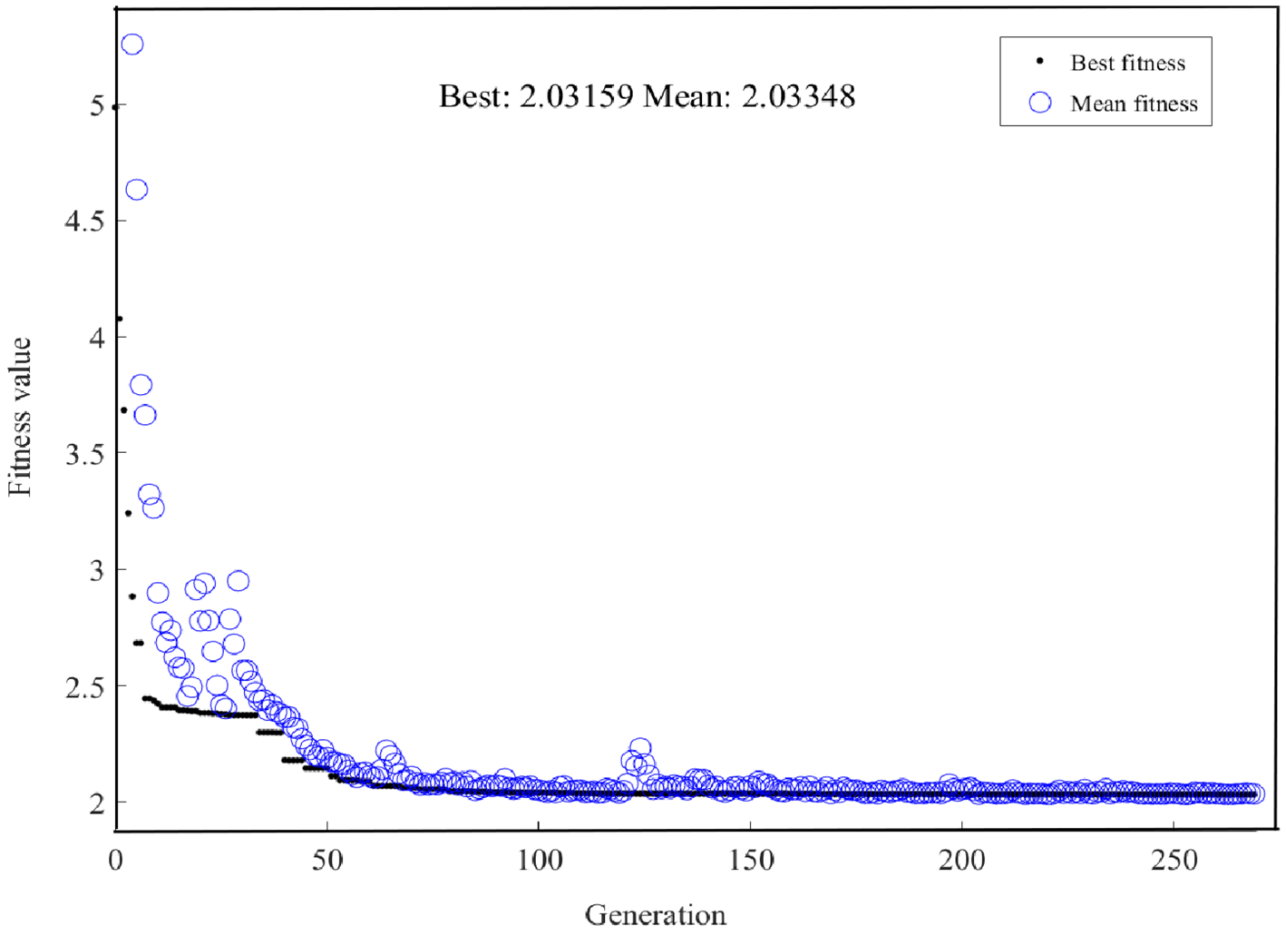
GA iterative graph for finding the minimum pulse duration.

After fitting through NNs, a GA is employed to identify the minimal value of pulse duration. In the case of locating the resonant cavity parameter settings corresponding to the minimum pulse duration, we randomly create a set of resonant cavity parameter within the range given in Table 1, input the NN and obtain the result of pulse duration, we use the seven cavity parameters and output pulse duration as the initial population of individuals. Then, we test these individuals and evaluate their fitness based on the nonlinear functions generated by the NN. Next, select the individual of minimum pulse duration with the highest score from the initial population as the parent of the gene, generate the next generation through crossover and mutation, and form a new population. Then, we compare the new generation of individuals with their parents, and if we find that the new generation of individuals is closer to the minimum pulse duration than the previous individuals, we stop calculating. In the end, we successfully find the minimum pulse duration and its corresponding cavity parameter. Figure 5 shows the performance of GA in progressively discovering the optimal state. It can be observed that as the number of evolutionary generations increases, the optimal value of the fitness function gradually decreases until it stabilizes at a minimum value. This indicates that the GA might have found a solution near the optimal solution in the search space. A comparison between the best fitness function value (Best) and the average fitness function value (Mean) curves serves as an estimation of the performance of GAs and the potency of the evolutionary process. If the Best curve is stable and close to the Mean curve, it indicates that the algorithm can find better solutions in the population and the search process is relatively balanced. Moreover, if the trend of fitness values ceases to improve or stabilizes with an increase in iterations, it might suggest that the algorithm has converged and halted evolving.

**Figure 6:**
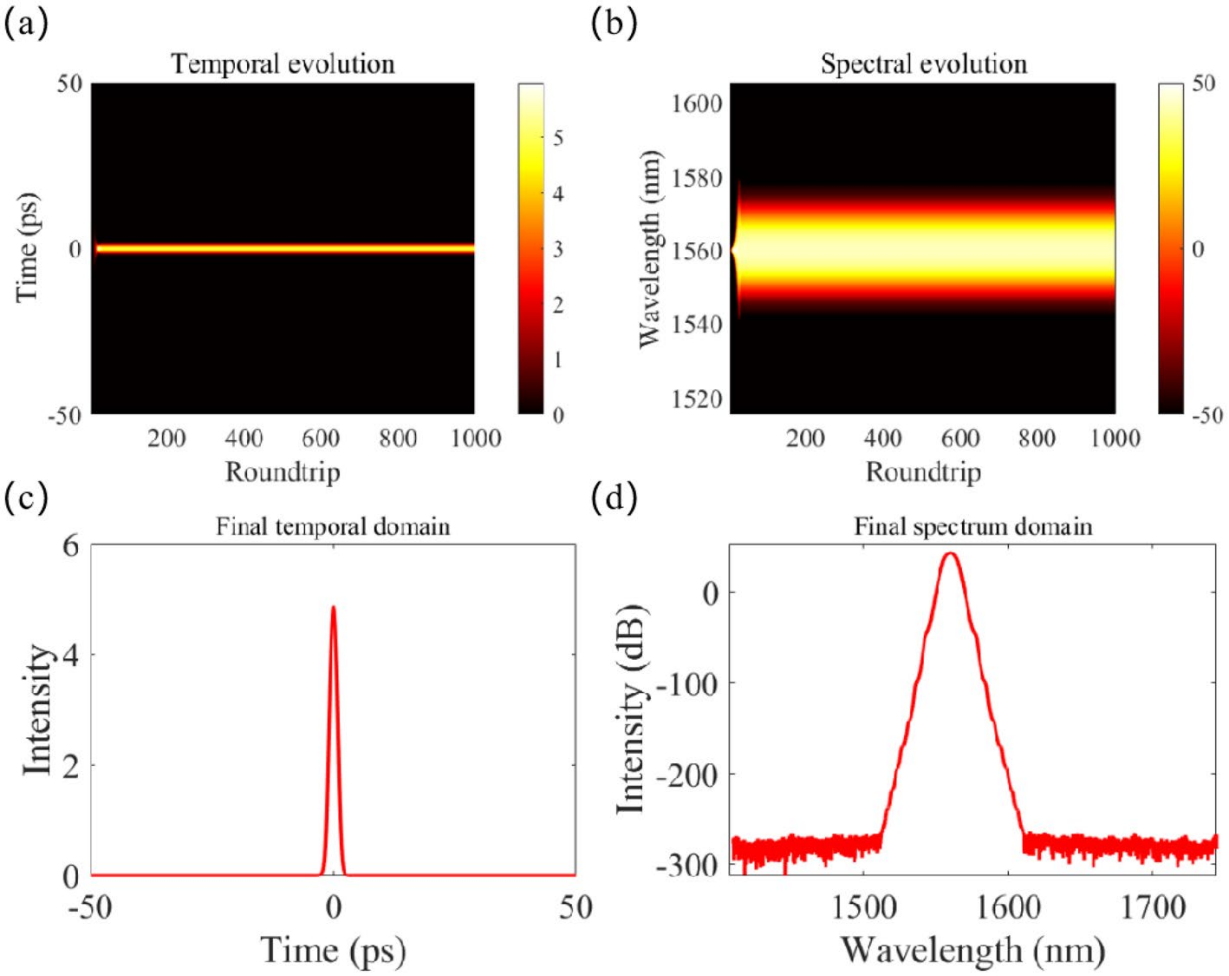
SSFT generation of minimum pulse duration. (**a**) temporal evolution of pulse in laser cavity; (**b**) spectral evolution of pulse in laser cavity; (**c**) stable temporal profile of pulse in laser cavity; (**d**) stable spectral profile of pulse in laser cavity.

The GA successfully succeeded in pinpointing the minimum pulse duration, predicted by the NN, to be 2.03159 ps. This coincided with the following seven laser cavity parameters: Ω = 4.529 × 10^–8^ nm, *E*_s_ = 4.2786 × 10^–11^ J, *γ*_EDF_ = 1 × 10^–3^/(W*m), *γ*_PF_ = 1 × 10^–3^/(W*m), *L*_0_ = 0.9, *P*_sat_ = 100 W, *g*_0_ = 1 dB/m. Figure 6 shows the ultrashort pulse’s parameter obtained by SSFT. The temporal evolution and spectral evolution of pulse in the laser cavity are depicted in Fig. 6(a), (b) respectively. The steady temporal and spectral pulse profiles within the laser cavity are reflected in Fig. 6(c), (d) respectively. The pulse duration, calculated by the SSFT method with the corresponding seven parameters, is simulated as 2.0269 ps. This implies a negligible relative difference in the pulse duration calculated by both methods and $\Delta = 2.31\%$.

**Figure 7:**
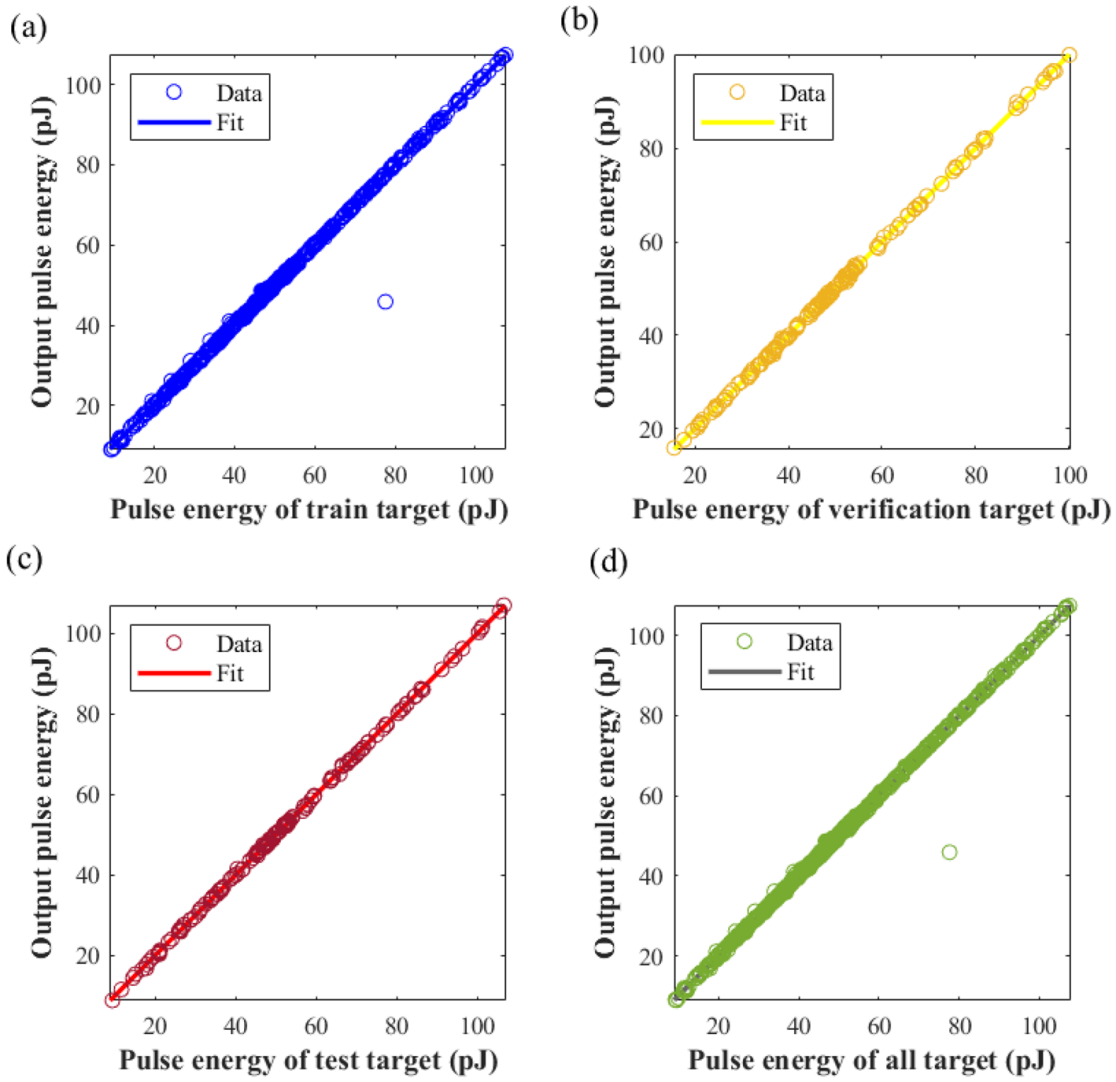
Comparative analysis of predicted and target values of pulse energy generated by NNs. (**a**) Training dataset regression diagram, (**b**) validation dataset regression diagram, (**c**) test dataset regression diagram, (**d**) regression diagram of all dataset.

For the second phase, the NN was adapted to fit the seven parameters of the laser cavity and pulse energy. Figure 7 displays the MSE derived from comparing the predicted and target values of energy produced by the second NNs. On fitting the NN, the MSE on the training set was found to be 1.5037, on the validation set it was 0.1024, on the test set at 0.0912, and on the entire dataset it scored 1.0816. As per Fig. 7, the regression coefficient of the training set is 99.79%, the validation set is 99.98%, the test set is 99.99%, and the entire dataset at 99.85%, indicating that the NN has excellent fitting of pulse energy.

**Figure 8:**
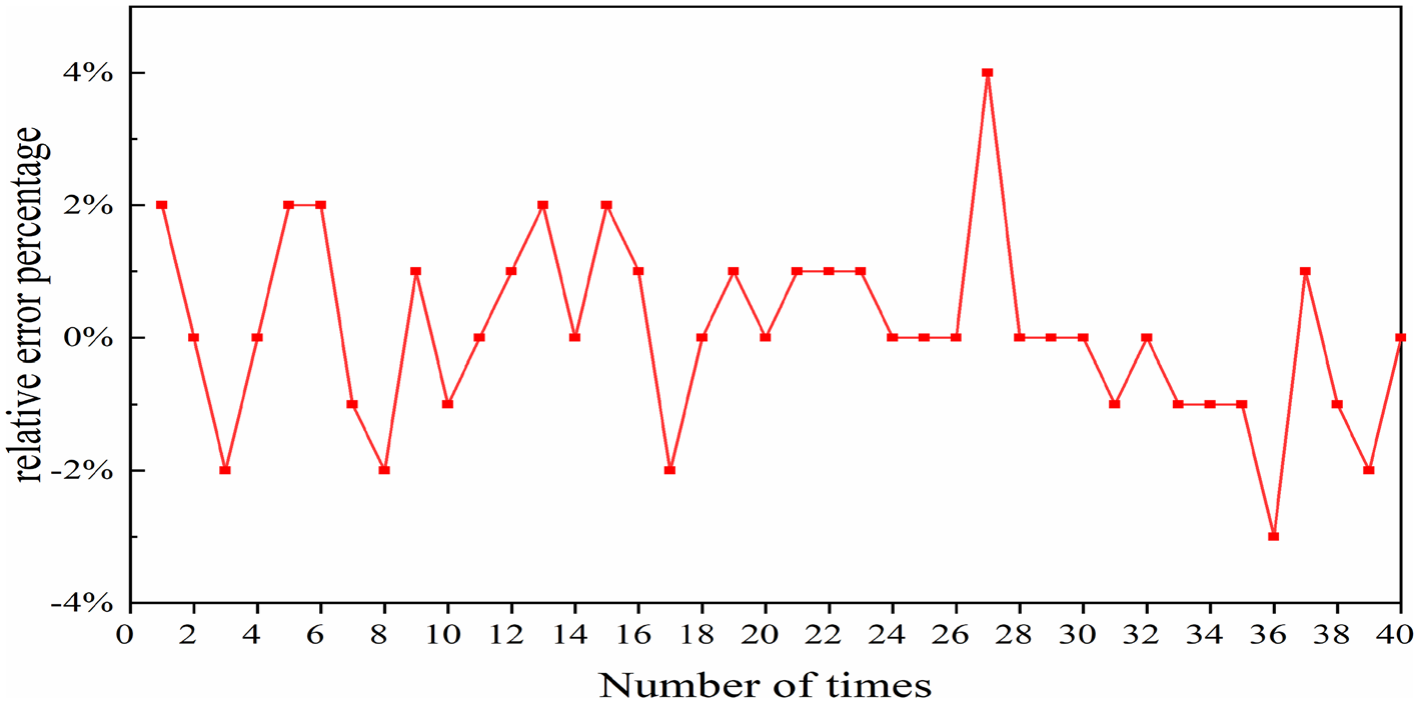
Relative percentage variation in pulse energy between the NNs and SSFT Over varied instances.

**Figure 9:**
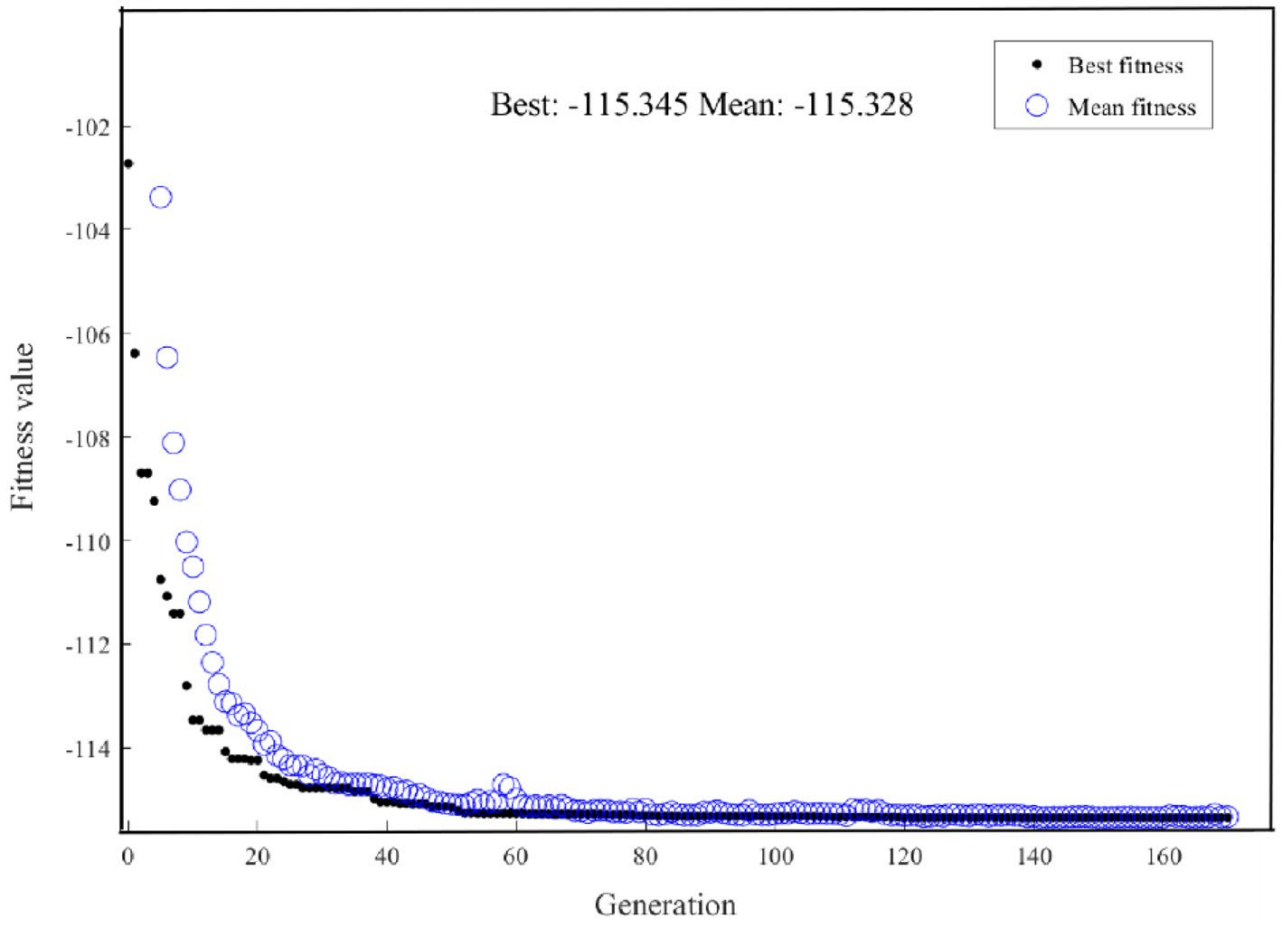
GA for finding the maximum predicted pulse energy iterative Graph.

To evaluate the difference between pulse energy values predicted by the NN and those obtained by using the SSFT method, we used 40 sets of randomly generated data. Figure 9 depicts the relative difference in pulse energy values computed by the two methods across these 40 sets. The calculation formula can be denoted as:

5 $$\Delta = \frac{{Q_{NN} - Q_{SSFT} }}{{Q_{SSFT} }} \times 100\% ,$$

where *Q*_NN_ is the energy predicted by the NN, *Q*_SSFT_ is the energy generated by SSFT. As demonstrated in Fig. 8, the relative error between the two methods peaks at 4% in the 27th set of data. This comparison suggests that the pulse energy predicted by the NN algorithm aligns reasonably well with the results of the SSFT method.

Figure 9 shows the performance of GA in progressively discovering the optimal state of pulse energy. It can be observed that as the number of evolutionary generations increases, the optimal value of the fitness function gradually decreases until it reaches a stable maximum value. If the fitness values plateau or cease to improve with increased iterations, it might suggest that the GA algorithm has converged and has ceased evolving. The maximum predicted pulse energy of 115.345 pJ was successfully located through GA, and in correspondence with the following seven parameters of laser cavity: *Ω* = 5.45 × 10^–8^ nm, *E*_s_ = 2 × 10^–10^ J, *γ*_EDF_ = 1 × 10^–3^/(W*m), *γ*_PF_ = 1.1 × 10^–3^/(W*m), *L*_0_ = 0.2, *P*_sat_ = 10 W, *g*_0_ = 10 dB/m. The pulse energy, computed by the SSFT method with the corresponding seven parameters, is recorded as 115.4176 pJ. This calculation leads to a negligible relative difference when compared to the results obtained by the two methods and $\Delta = - 0.06\%$.

**Figure 10:**
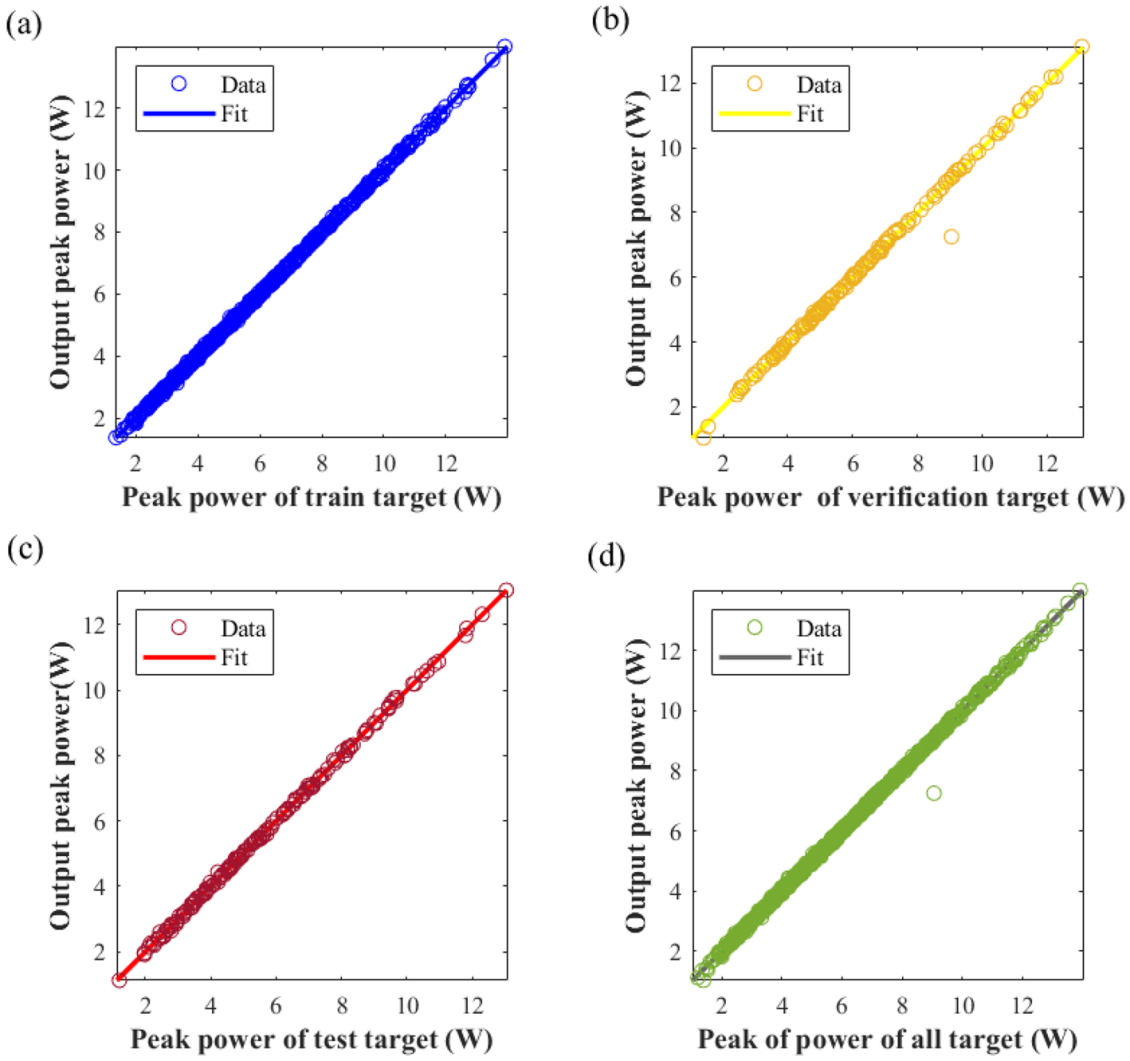
Comparative analysis of predicted and target values of peak power generated by NNs. (**a**) Training dataset regression diagram, (**b**) validation dataset regression diagram, (**c**) test dataset regression diagram, (**d**) regression diagram of all dataset.

The peak power of an ultrashort pulse in a laser can be described as:

6 $$P = \frac{Q}{\tau }.$$

where *P* represents the peak power of ultrashort pulses, *Q* represents the energy of the ultrashort pulse, and *τ* represents the pulse duration of the ultrashort pulse. The third NN was developed with the output layer representing the peak power of ultrashort pulse. The MSE of this model on the training set scored 0.002, on the validation set 0.024, on the test set 0.0029, and on the entire dataset 0.0054. Figure 10 shows the MSE of comparison between predicted and target values of peak power generated by the third NNs. It is evident that the correlation between the target value and the predicted value across all datasets surpasses 99%, with the fitting curve in each graph nearly coinciding with y = x. This indication suggests the successful fitting of the seven cavity parameters and peak power of the ultrashort pulse.

**Figure 11:**
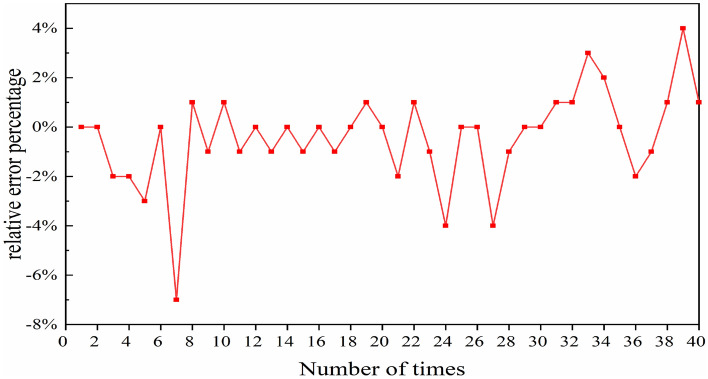
Relative percentage variation in peak power between NNs and SSFT over variable instances.

Similar to previous methods, Fig. 11 depicts the relative difference between pulse’s peak power as calculated by the two methods across 40 sets of randomly generated data points. The specific calculation formula is as follows:

7 $$\Delta = \frac{{P_{NN} - P_{SSFT} }}{{P_{SSFT} }} \times 100\% ,$$

where *P*_NN_ represents the peak power predicted by the NN, *P*_SSFT_ represents the peak power generated by SSFT. Reviewing Fig. 11, it is evident that all errors fluctuate between 0 and 7%. Particularly on the seventh set of data, the relative error peaks at 7%. This data corroborates the consistency between the peak power predicted by the NN algorithm and the results of the SSFT method.

**Figure 12:**
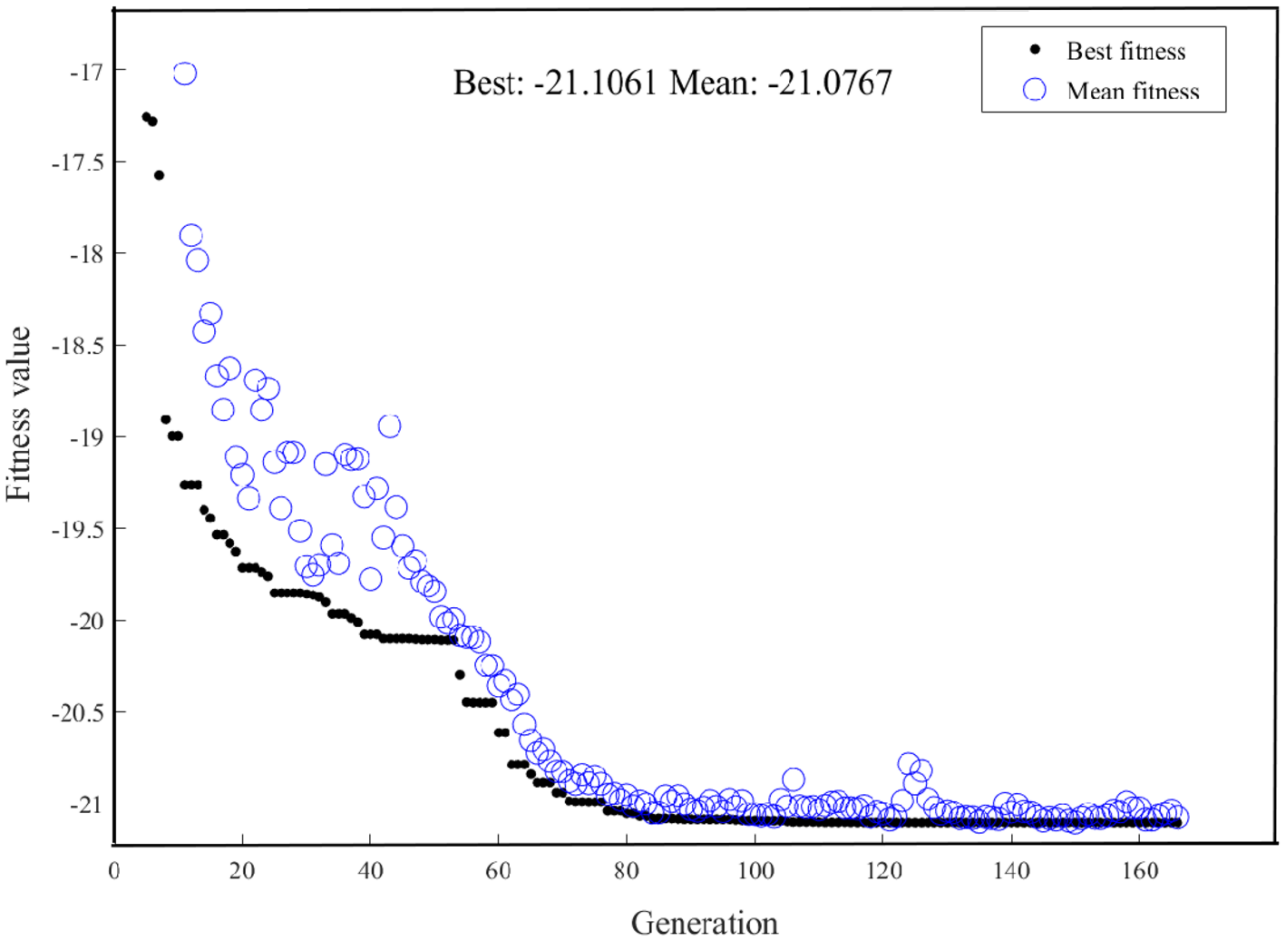
GA searching for maximum peak power iterative graph.

Figure 12 shows the performance of GA in increasingly identifying the optimal state of pulse’s peak power. As the number of evolutionary generations grows, the optimal value of the fitness function appears to diminish gradually until it stabilizes at a maximum value. GA successfully located the maximum predicted peak power of the pulse to be 21.1061 W, and the corresponding seven parameters of laser cavity are as follows: *Ω* = 2 × 10^–8^ nm, *E*_s_ = 2 × 10^–10^ J, *γ*_EDF_ = 5 × 10^–3^/(W*m), *γ*_PF_ = 5 × 10^–3^/(W*m), *L*_0_ = 0.9, *P*_sat_ = 100 W, *g*_0_ = 10 dB/m. The peak power of the pulse, calculated by the SSFT method with these corresponding parameters, is found to be 22.2118 W. The relative difference in the duration of the pulse obtained by two methods is a negligible and $\Delta = - 4.9\%$.

## Discussion

The intelligent algorithm has been designed to locate different pulse extremes—minimum pulse duration, maximum pulse energy, and maximum peak power—of fiber lasers. Through the assistance of AI algorithms for optimization, we have achieved significant improvements. The pulse width has been shortened to a minimum of 2.03159 ps, which is 0.96841 ps smaller than the minimum pulse width range in the dataset. The pulse energy has been increased, with a maximum recorded energy of 115.345 pJ, which exceeds the maximum energy range of 8.345 pJ in the dataset. The peak power has also been improved, with a maximum recorded peak power of 21.1061 W, exceeding the maximum power range of 7.1061 W in the dataset. The resonant cavity parameters corresponding to the optimal pulse parameters found through GA are shown in Table 2.

**Table 2:** Cavity parameters corresponding to the optimal pulse parameters.

Parameters	Pulse duration	Pulse energy	Peak power	Unit
*Ω*	4.529 × 10^–8^	5.45 × 10^–8^	2 × 10^–8^	nm
*E*_S_	4.2786 × 10^–11^	2 × 10^–10^	2 × 10^–10^	J
*γ*_EDF_	1 × 10^–3^	1 × 10^–3^	5 × 10^–3^	1/(W m)
*γ*_PF_	1 × 10^–3^	1.1 × 10^–3^	5 × 10^–3^	1/(W m)
*L*_0_	0.9	0.2	0.9	–
*P*sat	100	10	100	W
*g*_0_	1	10	10	dB/m

A cross-comparison of the results of seven laser cavity parameters reveals the following patterns: the gain bandwidth of EDF, nonlinear coefficient of EDF, and nonlinear coefficient of PF in the laser cavity parameters linked with minimum pulse duration and maximum pulse energy are strikingly similar—these parameters play a crucial role in the amplification and propagation of optical signals within the laser cavity. Also, the saturable power of SA and modulating depth of SA for minimum pulse duration and maximum peak power appear identical. The SA is a component that adjusts the amount of light absorption and thus significantly contributes to light intensity control. The small signal gain coefficient of EDF for maximum pulse energy and maximum peak power is also identical, affecting the gain of the pulse within the laser cavity.

During the optimization pulse process, the sample preparation time is approximately 4 h. The time for NN is about 5 s, the time for GA is about 10 s. So, the total time for intelligent algorithms is about 15 s. In GA, mutation rate and crossover rate are two key parameters that significantly affect the results and convergence efficiency of the algorithm. The mutation rate controls the probability of gene mutations, while the crossover rate regulates the probability of gene recombination. By finely adjusting these two parameters, we can optimize the search strategy and convergence speed of the algorithm. Here, we set the mutation rate and crossover rate to 0.4 and 0.5, respectively. Intelligent algorithms can quickly find the desired pulse, which means that once the sample is ready, the intelligent algorithm can quickly optimize and provide an optimize pulse. This kind of speed is very important for real-time applications or systems that require quick response.

It is very important to consider changes of external conditions in the application of intelligent algorithms and laser optimization processes. AI based optimization relies on a set of measured values. The actual values of the measured parameters depend on the ambient conditions of the corresponding laser, and the necessary measurement equipment must stay with the laser over its entire life time. Kobtsev et al*.* had raised questions about AI based optimization^28^. To ensure the stability of the external environment, a series of standard operating procedures should be set up, including regular calibration and environmental monitoring measures. From the software perspective, If the environmental factors that affect the laser output can be quantified, intelligent algorithms can also be used for environmental optimization, and adaptive algorithms can be written to adjust optimization parameters to ensure that the performance of the laser will not decrease due to environmental changes. In the case of SA, the modulation depth and saturable absorber power can be experimentally measured as a function of the laser’s operating time, a corresponding plan for the repeat rate of AI algorithm can be formulated. The laser parameters can be adjusted based on the variation pattern of its parameters with the help of AI algorithms, thereby achieving stable output of high-quality laser. Of course, these studies still need to be carried out, and many problems will be encountered during the implementation process, which is one aspect of this work's intended future research.

## Methods

**Figure 13:**
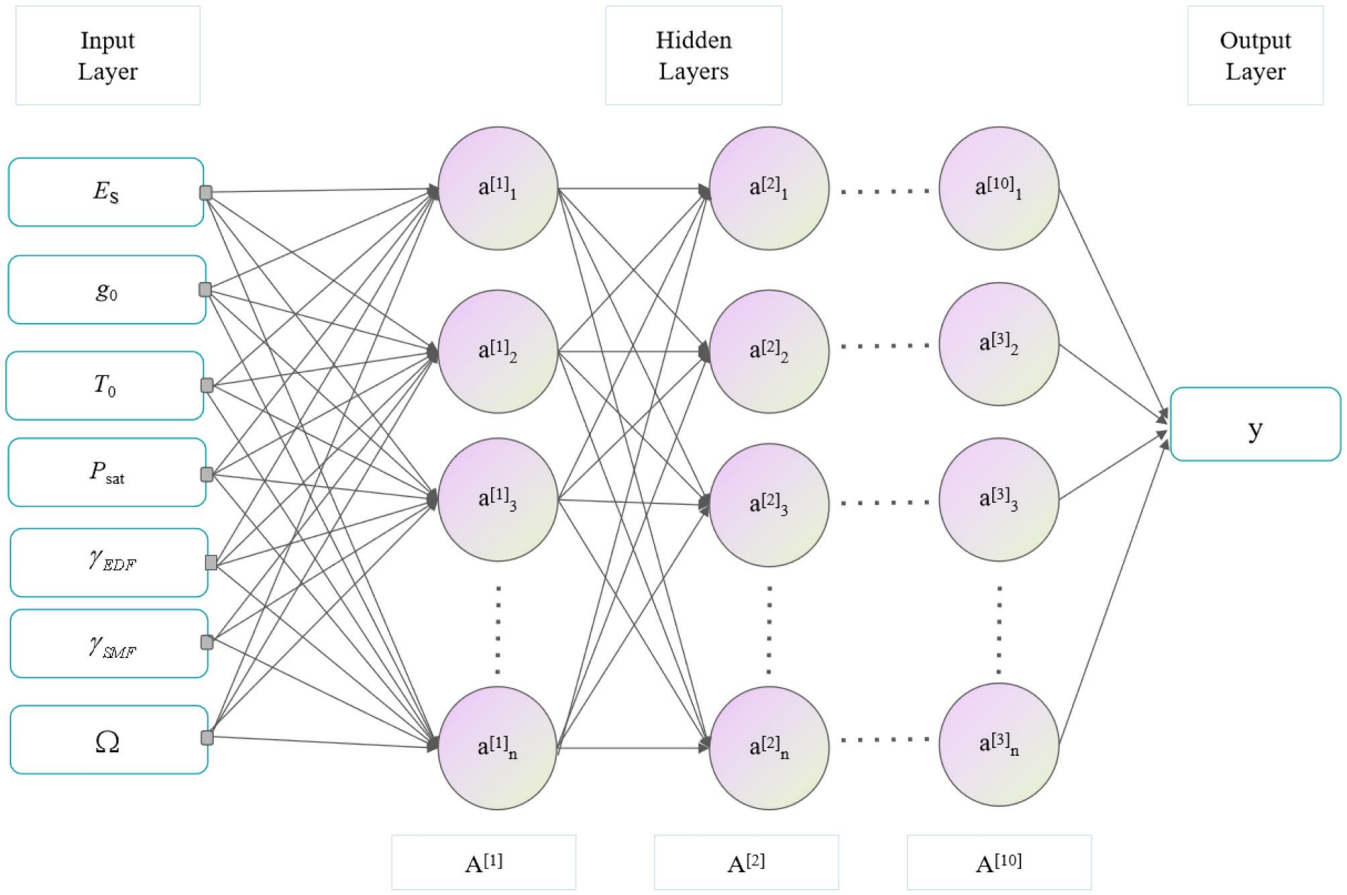
Relationship diagram between NN fitting laser parameters and ultrashort pulse indicators.

Understanding and mastering the relationship between laser parameters and the output characteristics are crucial in laser research and development, as these relationships often determine the performance and application of lasers. Therefore, the NN method employed herein can unravel the complex internal relationships between various parameters by learning from a large number of data samples. NNs, equipped with their multilayer structure and nonlinear activation functions, can capture and model highly nonlinear mapping relationships between input parameters and output characteristics. Once sufficient data samples are procured via the SSFT, we construct the NN illustrated in Fig. 13. The number of samples used here is 1000. This NN comprises an input layer (encasing seven laser parameters), 10 hidden layers, and an output layer corresponding to the laser's three output indicators: pulse duration, pulse energy, and peak power.

The NN is trained using the Levenberg Marquardt (LM) algorithm, The specific steps are as follows:1. Initialize NN parameter, which can be done using a random initialization method; 2. Employ forward propagation to calculate the NN’s output for a given input sample and obtain the predicted value; 3. Calculate the loss function using predicted values and actual labels. The Mean Squared Error (MSE) is typically used as the loss function; 4. Calculate the gradient using the backpropagation algorithm. This algorithm calculates the gradient of the loss function on network parameters, or the parameters' partial derivatives; 5. Deploy the LM algorithm to update network parameters. This minimizes the loss function by adjusting the parameters; 6. Check to see if the stop conditions have been met. These conditions could be reaching the maximum iterations or the loss function falling below a certain threshold; 7. Continue training or stop depending on whether the stop condition has been met.

In NNs, the LM algorithm is often employed to adjust the weights and biases of the network to minimize the sum of squared errors between the predicted output and the actual output. The weight increment’s calculation formula for the LM algorithm in NNs is as follows^29–32^

8 $$\Delta w = [J^{T} (w)J(w) + \mu I]^{ - 1} J^{T} (w)e(w),$$

where *w* is referred to as a vector consisting of the weights and thresholds of the neural NN, *I* is referred to as the identity matrix, *μ* is referred to as the learning rate, *J*(*w*) is referred to as the Jacobian matrix and *e*(*w*) is referred to as the error between the expected output value and the actual output value. The LM algorithm is an optimization algorithm that amalgamates the advantages of Gaussian Newton algorithm and gradient descent algorithm. When μ is small, the LM algorithm adopts the step size of the Gaussian Newton method, which is conducive to quickly converging to the local minimum in the parameter space; When μ is large, this method algorithm tends to use the step size of gradient descent to better cross local minima and find global minima.

## Data Availability

The datasets used and/or analyzed during the current study available from the corresponding author on reasonable request.

## References

[1] Fermann ME, Hartl I (2009). Ultrafast fiber laser technology. IEEE J. Sel. Top. Quantum Electron..

[2] Renninger WH, Wise FW (2013). Optical solitons in graded-index multimode fibres. Nat. Commun.

[3] Haus HA, Wong WJ (1996). Solitons in optical communications. Rev. Mod. Phys.

[4] Öktem B, Pavlov I, Ilday S, Kalaycıolu H, Rybak A, Yavas S, Erdoğan M, Ilday FÖ (2013). Nonlinear laser lithography for indefinitely large-area nanostructuring with femtosecond pulses. Nat. Photon..

[5] Grelu P, Akhmediev N (2012). Dissipative solitons for mode-locked lasers. Nat. Photon..

[6] Gattass R, Mazur E (2008). Femtosecond laser micromachining in transparent materials. Nat. Photon..

[7] Lecaplain C, Grelu Ph, Soto-Crespo JM, Akhmediev N (2012). Dissipative rogue waves generated by chaotic pulse bunching in a mode-locked laser. Phys. Rev. Lett..

[8] Peng JS, Boscolo S, Zhao Z, Zeng H (2019). Breathing dissipative solitons in mode-locked fiber lasers. Sci. Adv..

[9] Jordan MI, Mitchell TM (2015). Machine learning: Trends, perspectives, and prospects. Science.

[10] Jägersküpper J (2007). Algorithmic analysis of a basic evolutionary algorithm for continuous optimization. Theor. Comput. Sci..

[11] Genty G, Salmela L, Dudley JM, Brunner D, Kokhanovskiy A, Kobtsev S, Turitsyn SK (2021). Machine learning and applications in ultrafast photonics. Nat. Photon..

[12] Manish KA, Chet R (2017). A review on machine learning: Trends and future prospects. Res. Cell Int. J. Eng. Sci..

[13] Sung WT, Chiang YC (2012). Improved particle swarm optimization algorithm for android medical care IOT using modified parameters. J. Med. Syst..

[14] Almasinejad P, Golabpour A, Mollakhalili Meybodi MR, Mirzaie K, Khosravi A (2021). A dynamic model for imputing missing medical data: A multiobjective particle swarm optimization algorithm. J. Healthc. Eng..

[15] Dadheech P, Mehbodniya A, Tiwari S, Kumar S, Singh P, Gupta S, Atiglah HK (2022). Zika virus prediction using AI-driven technology and hybrid optimization algorithm in healthcare. J. Healthc. Eng..

[16] Sun Q, Wu QY (2022). Feature space fusion classification of remote sensing image based on ant colony optimisation algorithm. Int. J. Inf. Commun. Technol..

[17] Li W, Wozniak M (2022). A hole filling and optimization algorithm of remote sensing image based on bilateral filtering. Mobile Netw. Appl..

[18] Jegan L, Nandhitha N (2022). Design of optical filter using bald eagle search optimization algorithm. Intell. Autom. Soft Comput..

[19] Chang PC, Lin JJ, Liu CH (2012). An attribute weight assignment and particle swarm optimization algorithm for medical database classifications. Comput. Methods Programs Biomed..

[20] Jordan MI, Mitcheil TM (2015). Machine learning: Trends, perspectives, and prospects. Science.

[21] Herink G, Kurtz F, Jalali B, Solli DR, Ropers C (2017). Real-time spectral interferometry probes the internal dynamics of femtosecond soliton molecules. Science.

[22] Jiang M, Wu H, An Y (2022). Fiber laser development enabled by machine learning: Review and prospect. Photonix.

[23] Ma Q, Yu H (2023). artificial intelligence-enabled mode-locked fiber laser: A review. Nanomanuf. Metrol..

[24] Xu RQ, Xu FJ, Song YG, Duan L (2020). Impact of spectral filtering on pulse breaking-up and noise-like pulse generation in all-normal dispersion fiber lasers. Opt. Express.

[25] Picozzi A, Millot G, Wabnitz S (2015). Nonlinear virtues of multimode fibre. Nat. Photon..

[26] Pu GP, Yi LL, Zhang L, Hu WS (2020). Genetic algorithm-based fast real-time automatic mode-locked fiber laser. IEEE Photon. Technol. Lett..

[27] Woodward RI, Kelleher EJR (2016). Towards ‘smart lasers’: Self-optimisation of an ultrafast pulse source using a genetic algorithm. Sci. Rep..

[28] Kobtsev SM (2021). Perspective paper: Can machine learning become a universal method of laser photonics?. Opt. Fiber Technol..

[29] Willamowski BM, Yu H (2010). Improved computation for Levenberg–Marquardt training. IEEE Trans. Neural Netw..

[30] Rubio JDJ (2021). Stability analysis of the modified Levenberg–Marquardt algorithm for the artificial neural network training. IEEE Trans. Neural Netw. Learn. Syst..

[31] Wang RC, Wu S (2019). Neural network model based prediction of fragmentation of blasting using the Levenberg–Marquardt algorithm. J. Hydroelectr. Eng..

[32] Liu ZM, Du SQ, Wang RY (2018). Levenberg Marquardt algorithm for solving linear complementarity problems. J. Appl. Math..

